# The global prevalence and associated risk factors of *Eimeria* infection in domestic chickens: A systematic review and meta‐analysis

**DOI:** 10.1002/vms3.1469

**Published:** 2024-05-30

**Authors:** Milad Badri, Meysam Olfatifar, Alireza Hayati, Behzad Bijani, Rasoul Samimi, Amir Abdoli, Oskar Nowak, Daniel Diaz, Aida Vafae Eslahi

**Affiliations:** ^1^ Medical Microbiology Research Center Qazvin University of Medical Sciences Qazvin Iran; ^2^ Gastroenterology and Hepatology Diseases Research Center Qom University of Medical Sciences Qom Iran; ^3^ Students' Research Committee (SRC) Qazvin University of Medical Sciences Qazvin Iran; ^4^ Zoonoses Research Center Jahrom University of Medical Sciences Jahrom Iran; ^5^ Department of Parasitology and Mycology Jahrom University of Medical Sciences Jahrom Iran; ^6^ Institute of Human Biology and Evolution Faculty of Biology Adam Mickiewicz University Poznań Poland; ^7^ Facultad de Ciencias Universidad Nacional Autónoma de México, Copilco Ciudad de México México

**Keywords:** *Eimeria* infection, domestic chicken, prevalence, meta‐analysis, worldwide

## Abstract

**Background:**

*Eimeria* is a protozoan parasite that affects poultry, particularly chickens, causing a disease known as coccidiosis. This disease imposes substantial significant economic challenges to the poultry sector.

**Objectives:**

The current study aimed to estimate the global prevalence and associated risk factors of *Eimeria* in domestic chickens.

**Methods:**

Multiple databases (Scopus, PubMed, ProQuest, Web of Science and Google Scholar) were searched for articles published until June 2023. The pooled prevalence was estimated using a random‐effects model with a 95% confidence interval. The statistical analysis was conducted using meta packages in R version (3.6.1).

**Results:**

In total, 41 articles fulfilled the eligibility criteria. The global pooled prevalence was 44.3% (36.9%–51.8%) with *Eimeria tenella* (38.7%, 30.1%–47.7%) as the most prevalent species. The highest pooled prevalence was related to the Western Pacific Region (80.5%, 72.6%–87.3%) and urban areas (44.4%, 36.5%–52.6%). Moreover, areas with humid subtropical climates represent the highest overall prevalence (75.8%, 46.6%–95.9%).

**Conclusion:**

The necessity for robust and innovative strategies for preventing and managing this disease cannot be overstated. Addressing *Eimeria* impact is crucial not only for safeguarding poultry health but also for sustaining the economic viability of the poultry industry.

## Graphical abstract

Coccidiosis caused by *Eimeria* parasites is a serious disease in chickens. It leads to diarrhoea, weight loss, decreased egg production and even death in severe cases. It poses substantial economic conflictions to the poultry sector. Preventing and managing this disease in poultry through a robust and innovative strategy is imperative. Regular monitoring and early intervention, along with proper hygiene, vaccination and use of medications, are essential to minimize the impact of the disease in chicken populations.

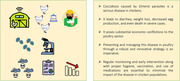

## 1 INTRODUCTION

Coccidiosis is recognized as the parasitic disease with the highest economic impact on the poultry production industry (Allen & Fetterer, 2002). A wide range of domestic and wild animal species, including cattle, sheep, goats, pigs, chickens, turkeys, rabbits, reptiles and fish, are affected by coccidiosis, which is the most potent cause of economic losses, especially regarding poultry and livestock farming (Ahmad et al., 2016; Jenkins, 2001). The disease affects chickens, with infection rates reaching 5% and 20% for clinical and sub‐clinical cases, respectively. The financial impact of coccidiosis is considerable, estimated to be between 2.4 and 3 billion USD annually (Martin et al., 1995; Qaid et al., 2021; Shirley et al., 2005; Zhang et al., 2013).

The principal causative agents of coccidiosis in poultry are attributed to members of the *Eimeria* genus, which are obligatory intracellular protozoan parasites of the apicomplexan class, classified within the family *Eimeriidae* (Peek & others, 2010; Shirley et al., 2005). In recent years, concerns over the impact of infections caused by *Eimeria* species on the livestock and poultry industries have gained increasing attention on a global scale (Almeria et al., 2020; Kadykalo et al., 2018; Peek & Landman, 2011).

There are seven different species of *Eimeria* in chickens that mature within the intestinal epithelial cells of the definitive host (Blake & Tomley, 2014; Jenkins, 2001; McDonald & Shirley, 2009). Among different species, *Eimeria tenella*, *Eimeria acervulina* and *Eimeria maxima* impose substantial economic losses in poultry industry (El‐Ghany, 2020).

*Eimeria* parasites have exogenous and endogenous life cycle. The exogenous phase involves the release of oocysts into the environment, whereas the endogenous phase occurs within the host's intestine, where the parasite undergoes asexual (schizogony) and sexual reproduction (gametogeny) (McDonald & Shirley, 2009). The infection can be potentially transmitted through various routes, such as direct bird‐to‐bird contact, clothing and footwear, insects like flies and beetles, as well as wild birds that come into contact with poultry facilities, breeding equipment and non‐sterilized contaminated feed bags. In addition, climatic conditions have a significant impact on transmission, as warm and humid tropical climates are potential contributors to the occurrence of these parasites (Abdelrahman et al., 2014; Etuk et al., 2004; Serbessa et al., 2023; Toledo et al., 2011).

The infection is commonly associated with severe intestinal conflicts, diarrhoea, dehydration, weight loss, lack of appetite and weakness. It significantly impacts animal farming by reducing production and increasing mortality rates (Boulton et al., 2018; El‐Shazly et al., 2020; Zhou et al., 2020b).

Adopting effective methods of caring for animals and using preventive treatments like various types of anticoccidial medications (such as sulphonamides, ionophores and toltrazuril) that work against different parts of the parasite's life cycle are the recommended approaches to avoid the disease, especially in the field of poultry farming (Chartier & Paraud, 2012; McDonald & Shirley, 2009).

The current study aims to enhance our understanding of the global prevalence of *Eimeria* parasites in chickens by thoroughly analysing the available studies, which help to improve practices concerning poultry care and provide valuable insights for future research.

## 2 MATERIALS AND METHODS

### 2.1 Search strategy

This study complies with the preferred reporting items for systematic reviews and meta‐analysis checklist (Page et al., 2021). We conducted searches across multiple databases (Scopus, PubMed, ProQuest, Web of Science and Google Scholar) to retrieve papers published up to June 2023 without a lower date limit. Search terms were as follows; prevalence, incidence, proportion, domestic bird, domestic poultry, poultry diseases, protozoal infections, protozoan parasites, coccidiosis, *E. tenella*, *E. acervulina*, *Eimeria necatrix*, *E. maxima*, *Eimeria mitis*, *Eimeria brunetti*, *Eimeria praecox Eimeria mivati* are worldwide and global, using Boolean operators (AND, OR). Duplicate papers were automatically removed through the utilization of EndNote software X9 version. Additionally, the reference list was manually searched to identify additional relevant studies that were not retrievable through a database search. Two authors independently conducted searches, assessed titles and abstracts, and reviewed the full texts of the papers.

### 2.2 Inclusion and exclusion criteria

Full‐text articles were considered eligible if they satisfied the inclusion criteria outlined as follows:

1. Cross‐sectional studies reporting the *Eimeria* parasites in domestic chicken
2. Peer‐reviewed original articles
3. Availability of full‐text and abstract in English
4. Availability of total sample size and the exact number of positive subjects

The present study excluded the following types of papers from analysis: case series, case reports, letters, editorials, non‐original data publications, review articles, papers with unclear or equivocal findings, non‐English language papers and papers reporting protozoan parasites in samples related to subjects other than domestic chickens. Microsoft Excel version 2016 was used to independently collect the following information from the eligible papers: author's name, year of publication, climate, annual precipitation, annual rainfall, humidity, average temperature, seasons, WHO region, species of *Eimeria*, type of region (rural/urban), sample type and diagnostic method (Tables 1 & 2).

**TABLE 1:** Main characteristics of the included studies reporting the prevalence of *Eimeria* in domestic chicken.

Study no.	Author	Year	Country	Continent	Sample size	Infected	Type of *Eimeria*	Reference
1	Razmia and Kalideri	2000	Iran	Asia	84	32.00	*E. acervulina*	Razmi and Kalideri (2000)
							*E. tenella*	
							*E. maxima*	
2	Al‐Natour et al.	2002	Jordan	Asia	200	155	*E. acervulina*	Al‐Natour et al. (2002)
							*E. tenella*	
							*E. mitis*	
							*E. maxima*	
							*E. necatrix*	
							*E. brunetti*	
							*E. mivati*	
3	Lobago et al.	2005	Ethiopia	Africa	965	370	*E. acervulina*	Lobago et al. (2005)
							*E. tenella*	
							*E. necatrix*	
							*E. brunetti*	
4	Mungube et al.	2008	Kenya	Africa	360	84	*E. tenella*	Mungube et al. (2008)
							*E. necatrix*	
5	Nematollahi et al.	2009	Iran	Asia	218	122	*E. acervulina*	Nematollahi et al. (2009)
							*E. tenella*	
							*E. mitis*	
							*E. maxima*	
							*E. necatrix*	
6	Aarthi et al.	2010	India	Asia	43	25	*E. acervulina*	Aarthi et al. (2010)
							*E. brunetti*	
							*E. tenella*	
							*E. mitis*	
							*E. praecox*	
							*E. maxima*	
							*E. necatrix*	
7	Lee et al.	2010	South Korea	Asia	356	280	*E. acervulina*	Lee et al. (2010)
							*E. brunetti*	
							*E. tenella*	
							*E. mitis*	
							*E. praecox*	
							*E. maxima*	
							*E. necatrix*	
8	Hadipour et al.	2011	Iran	Asia	200	128	*E. acervulina*	Hadipour et al. (2011)
							*E. tenella*	
							*E. maxima*	
							*E. necatrix*	
9	Shirzad et al.	2011	Iran	Asia	120	90	*E. acervulina*	Shirzad et al. (2011)
							*E. tenella*	
							*E. maxima*	
							*E. necatrix*	
							*E. brunetti*	
10	Awais et al.	2012	Pakistan	Asia	7480	3283	*E. acervulina*	Awais et al. (2012)
							*E. tenella*	
							*E. maxima*	
							*E. necatrix*	
11	Györke et al.	2013	Romania	Europe	23	21	*E. acervulina*	Györke et al. (2013)
							*E. tenella*	
							*E. praecox*	
							*E. maxima*	
12	Al Se et al.	2013	Iraq	Asia	129	28	*E. acervulina*	Al Se et al. (2013)
							*E. tenella*	
							*E. mitis*	
							*E. maxima*	
							*E. necatrix*	
							*E. brunetti*	
13	Luu et al.	2013	Ethiopia	Africa	767	427	*E. acervulina*	Luu et al. (2013)
							*E. tenella*	
							*E. mitis*	
							*E. praecox*	
							*E. maxima*	
							*E. necatrix*	
							*E. brunetti*	
14	Sharma et al.	2013	India	Asia	720	285	*E. necatrix*	Sharma et al. (2013)
							*E. maxima*	
							*E. acervulina*	
							*E. mitis*	
							*E. tenella*	
15	Gharekhani et al.	2014	Iran	Africa	220	70	*E. acervulina*	Gharekhani et al. (2014)
							*E. tenella*	
							*E. necatrix*	
							*E. maxima*	
16	Olanrewaju and Agbor	2014	Nigeria	Africa	200	138	*E. acervulina*	Olanrewaju and Agbor (2014)
							*E. tenella*	
							*E. maxima*	
17	Sharma et al.	2015	India	Asia	240	48	*E. acervulina*	Sharma et al. (2015)
							*E. tenella*	
							*E. mitis*	
							*E. necatrix*	
							*E. maxima*	
18	Garbi et al.	2015	Ethiopia	Africa	384	75	*E. acervulina*	Garbi et al. (2015)
							*E. tenella*	
							*E. mitis*	
							*E. maxima*	
							*E. necatrix*	
							*E. brunetti*	
19	Mokhtar and Yagoob	2016	Iran	Asia	384	135	*Eimeria spp*.	Mokhtar and Yagoob (2016)
20	Kaboudi et al.	2016	Tunisia	Africa	630	200	*E. acervulina*	Kaboudi et al. (2016)
							*E. tenella*	
							*E. maxima*	
21	Jamil and Mansoor	2016	Pakistan	Asia	300	132	*E. tenella*	Jamil and Mansoor (2016)
							*E. maxima*	
							*E. necatrix*	
							*E. mitis*	
22	Yakhchali and Fakhri	2017	Iran	Asia	130	28	*E. tenella*	Yakhchali and Fakhri (2017)
							*E. maxima*	
							*E. necatrix*	
							*E. acervulina*	
23	Huang et al.	2017	China	Asia	171	150	*E. acervulina*	Huang et al. (2017)
							*E. tenella*	
							*E. mitis*	
							*E. maxima*	
							*E. necatrix*	
							*E. brunetti*	
24	Debbou‐Iouknane et al.	2018	Algeria	Africa	109	78	*E. acervulina*	Debbou‐Iouknane et al. (2018)
							*E. tenella*	
							*E. maxima*	
							*E. brunetti*	
							*E. mitis*	
25	Yousaf et al.	2018	Pakistan	Asia	420	100	*Eimeria spp*.	Yousaf et al. (2018)
26	Hamza et al.	2018	Iraq	Asia	200	160	*E. tenella*	Hamza et al. (2018)
							*E. maxima*	
							*E. necatrix*	
27	Ghasemian et al.	2019	Iran	Asia	100	15	*E. acervulina*	Ghasemian et al. (2019)
							*E. tenella*	
							*E. mitis*	
							*E. brunetti*	
28	Montes‐Vergara et al.	2021	Colombia	South America	535	362	*Eimeria spp*.	Montes‐Vergara et al. (2021)
29	Bawm et al.	2021	Myanmar	Asia	122	41	*E. praecox*	Bawm et al. (2021)
							*E. maxima*	
30	Das	2021	India	Asia	674	203	*E. acervulina*	Das (2021)
							*E. tenella*	
							*E. mitis*	
							*E. praecox*	
							*E. maxima*	
							*E. necatrix*	
							*E. brunetti*	
							*E. mivati*	
31	Carrisosa et al.	2021	United States	North America	82	50	*Eimeria spp*.	Carrisosa et al. (2021)
32	Akanbi et al.	2022	Nigeria	Africa	1843	207	*Eimeria spp*.	Akanbi et al. (2022)
33	Auwal et al.	2022	Nigeria	Africa	384	103	*E. acervulina*	Auwal et al. (2022)
							*E. tenella*	
							*E. maxima*	
							*E. necatrix*	
34	Jan et al.	2022	India	Asia	780	293	*E. tenella*	Jan et al. (2022)
							*E. acervulina*	
							*E. maxima*	
							*E. necatrix*	
							*E. mitis*	
35	Khursheed et al.	2022	India	Asia	600	171	*E. acervulina*	Khursheed et al. (2022)
							*E. necatrix*	
							*E. maxima*	
							*E. tenella*	
36	Flores et al.	2022	South Korea	Asia	388	291	*E. acervulina*	Flores et al. (2022)
							*E. maxima*	
							*E. tenella*	
							*E. mitis*	
							*E. praecox*	
							*E. brunetti*	
							*E. necatrix*	
37	Nana‐Mariam et al.	2023	Nigeria	Africa	204	74	*E. acervulina*	Nana‐Mariam et al. (2023)
							*E. necatrix*	
							*E. brunetti*	
							*E. tenella*	
							*E. mitis*	
38	Adem et al.	2023	Ethiopia	Africa	450	122	*Eimeria spp*.	Adem et al. (2023)
39	Yaqub et al.	2023	India	Asia	3057	325	*E. tenella*	Yaqub et al. (2023)
							*E. acervulina*	
							*E. maxima*	
							*E. necatrix*	
							*E. mitis*	
							*E. praecox*	
							*E. brunetti*	
40	Mares et al.	2023	Saudi Arabia	Asia	120	30	*E. tenella*	Mares et al. (2023)
							*E. necatrix*	
							*E. acervulina*	
							*E. maxima*	
							*E. praecox*	
41	Pajic et al.	2023	Serbia	Europe	100	59	*E. acervulina*	Pajić et al. (2023)
							*E. maxima*	
							*E. mitis*	
							*E. tenella*	

**TABLE 2:** Sub‐group analysis based on climate, seasons, diagnostic method, humidity, longitude, latitude, sample type, type of region (rural/urban), WHO regions, annual precipitation, annual rainfall and average temperature in included studies.

					Heterogeneity		
Variables	No studies	Sample size	Infected	Pooled prevalence (95%CI)	*I* ^2^ (%)	*τ* ^2^ (%)	*p*‐Value
**Climate**							
Tropical savanna climate	17	10,234	2899	36.73% (27.3%–46.6%)	99	4.3	<0.001
Hot semi‐arid climate	1	630	200	31.7% (28.2%–35.5%)			<0.001
Hot desert climate	9	10,801	4173	43.6% (26.1%–61.9%)	99	7.8	<0.001
Cold semi‐arid climate	7	1072	485	42.5% (25.9%–60.0%)	97	5.4	<0.001
Humid continental climate	3	844	630	71.8% (60.1%–82.2%)	86	1.1	<0.001
Humid subtropical climate	2	253	200	75.8% (46.6%–95.9%)	95	4.5	<0.001
Tropical monsoon climate	1	122	41	33.6% (25.8%–42.4%)	–	–	–
Tropical rainforest climate	1	535	362	67.6% (63.9%–71.9%)	–	–	–
**Seasons**							
Spring	11	4302	924	40.8% (26.6%–55.8%)	98	6.0	<0.001
Summer	10	1558	515	38.3% (21.4%–56.7%)	96	8.1	<0.001
Autumn	12	2581	1254	58.6% (40.5%–75.6%)	98	9.8	<0.001
Winter	11	1466	633	41.2% (27.4%–55.6%)	96	5.6	<0.001
**Diagnostic method**							
Parasitology	29	21,520	7285	37.4% (30.1%–44.5%)	99	4.3	<0.001
PCR + Parasitology	7	2121	1140	63.2% (43.2%–81.1%)	98	7.0	<0.001
PCR	7	1078	762	68.2% (50.9%–83.1%)	96	5.3	<0.001
**Humidity**							
<40	21	8402	2562	46.7% (35.1%–58.5%)	99	7.3	<0.001
>75	7	3378	1303	40.9% (28.7%–53.7%)	97	2.9	<0.001
40–75	13	12,712	5125	42.4% (30%–55.5%)	99	5.6	<0.001
**Latitude**							
<20	10	6092	1962	36.5% (24.3%–49.8%)	99	4.6	<0.001
>50	1	171	150	87.7% (82.0%–91.9%)			–
20–35	14	7331	1978	41.5% (29.9%–53.5%)	99	5.1	<0.001
36–50	16	10,898	4900	48.9% (36.6%–61.2%)	98	6.2	<0.001
**Longitude**							
<10	5	2740	600	41.6% (18.9%–66.4%)	99	8.0	<0.001
>110	2	744	571	76.8% (72.9%–80.5%)	27	0.0	<0.001
10–60	20	5784	2351	43.8% (33.2%–54.6%)	97	5.9	<0.001
61–110	14	15,224	5468	41% (29.8%–53.0%)	99	5.0	<0.001
**Sample type**							
Necropsy	15	15,851	5230	40.3% (29.4%–51.7%)	99	4.9	<0.001
Stool	25	8421	3690	47.3% (37.2%–57.5%)	98	6.6	<0.001
Stool and necropsy	1	220	70	31.8% (26.0%–38.2%)	–	–	–
**Type of region (rural/urban)**							
Rural	4	1219	624	43.5% (24.9%–63.1%)	96	3.9	<0.001
Urban	37	23,273	8366	44.4% (36.5%–52.6%)	99	6.2	<0.001
**WHO regions**							
African Region	11	6296	1878	36.3% (24.8%–48.6%)	99	4.4	<0.001
Eastern Mediterranean Region	15	10,305	4508	43.2% (31.8%–55.0%)	97	5.2	<0.001
European Region	2	123	80	75.7% (41.2%–98.1%)	90	5.7	<0.001
Region Of Americans	2	617	412	66.0% (59.6%–72.0%)	30	0.1	<0.001
South‐East Asia Region	8	6236	1391	30.8% (21.4%–41.0%)	99	2.2	<0.001
Western Pacific Region	3	915	721	80.5% (72.6%–87.3%)	84	0.5	<0.001
**Annual precipitation**							
<300	14	2844	1271	45.8% (31.9%–60.1%)	97	6.0	<0.001
>1000	16	10,228	2896	41.8% (29.4%–54.7%)	99	5.7	<0.001
300–650	7	8854	3829	53.6% (26.5%–79.5%)	98	8.7	<0.001
651–1000	4	2566	994	34.7% (12.7%–60.8%)	98	2.7	<0.001
**Annual rainfall**							
<400	8	9564	4074	41.0% (26.1%–56.9%)	97	0.5	<0.001
>1500	2	165	66	44.9% (22.9%–67.9%)	87	2.4	<0.001
1001–1500	21	9398	3719	45.7% (36.2%–55.4%)	99	5.0	<0.001
401–1000	10	5365	1131	44.3% (24.8%–64.7%)	99	1.1	<0.001
**Average temperature**							
<10	2	3657	496	18.7% (4.8%–38.8%)	99	2.6	<0.001
>20	17	14,267	5476	36.7% (27.8%–46.2%)	99	3.9	<0.001
10–20	22	6568	3018	53.0% (42.6%–63.3%)	98	6.0	<0.001

Abbreviations: CI, confidence interval; PCR, polymerase chain reaction.

### 2.3 Quality assessment

The assessment of study quality was carried out through the utilization of the Newcastle‐Ottawa Scale (as described in Table S1) (Abdoli et al., 2024; Eslahi et al., 2023). The scoring process included three components: (1) selection (maximum of 5 stars), (2) comparability (maximum of 2 stars) and (3) outcome (maximum of 3 stars), each with its respective score ranges.

### 2.4 Data synthesis and statistical analysis

To estimate the pooled prevalence of *Eimeria* parasites in domestic chickens, a Freeman–Tukey double arcsine transformation was applied using a random‐effects model with a 95% confidence interval (95%CI).

In order to specify the impact of the year of publication on the prevalence, a meta‐regression analysis was applied.

We employed Egger's and Begg's tests to identify potential publication bias. Additionally, publication bias was assessed using the Luis Furuya–Kanamori (LFK) index and the Doi plot (Barendregt & Doi, 2016). An LFK index within the range of outside ±2, ±2 and ±1 indicated significantly/major asymmetrical, slightly/minor asymmetrical and asymmetrical symmetrical (absence of publication bias), respectively. Furthermore, we assessed the degree of heterogeneity among the included studies using Cochrane's *Q* test and the inconsistency index (*I* ^2^ statistics), considering *I* ^2^ values of 0%–25% as low, 25%–50% as moderate and 50%–75% as high heterogeneity (Higgins et al., 2003). A *p*‐value less than 0.05 was specified as statistically significant. All procedures of statistical analyses were performed via meta and metasens packages in R version (3.6.1) (R Core Team, 2020).

## 3 RESULTS

### 3.1 Characteristics of included studies

**FIGURE 1:**
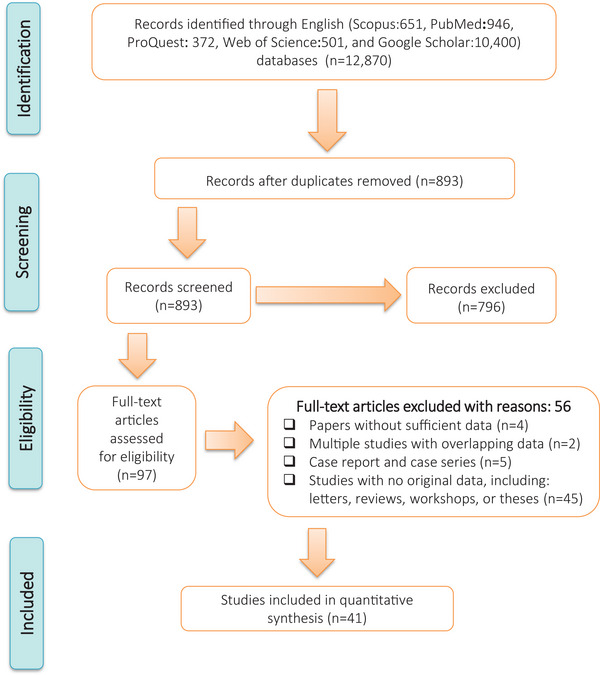
Flow diagram of the study design process.

**FIGURE 2:**
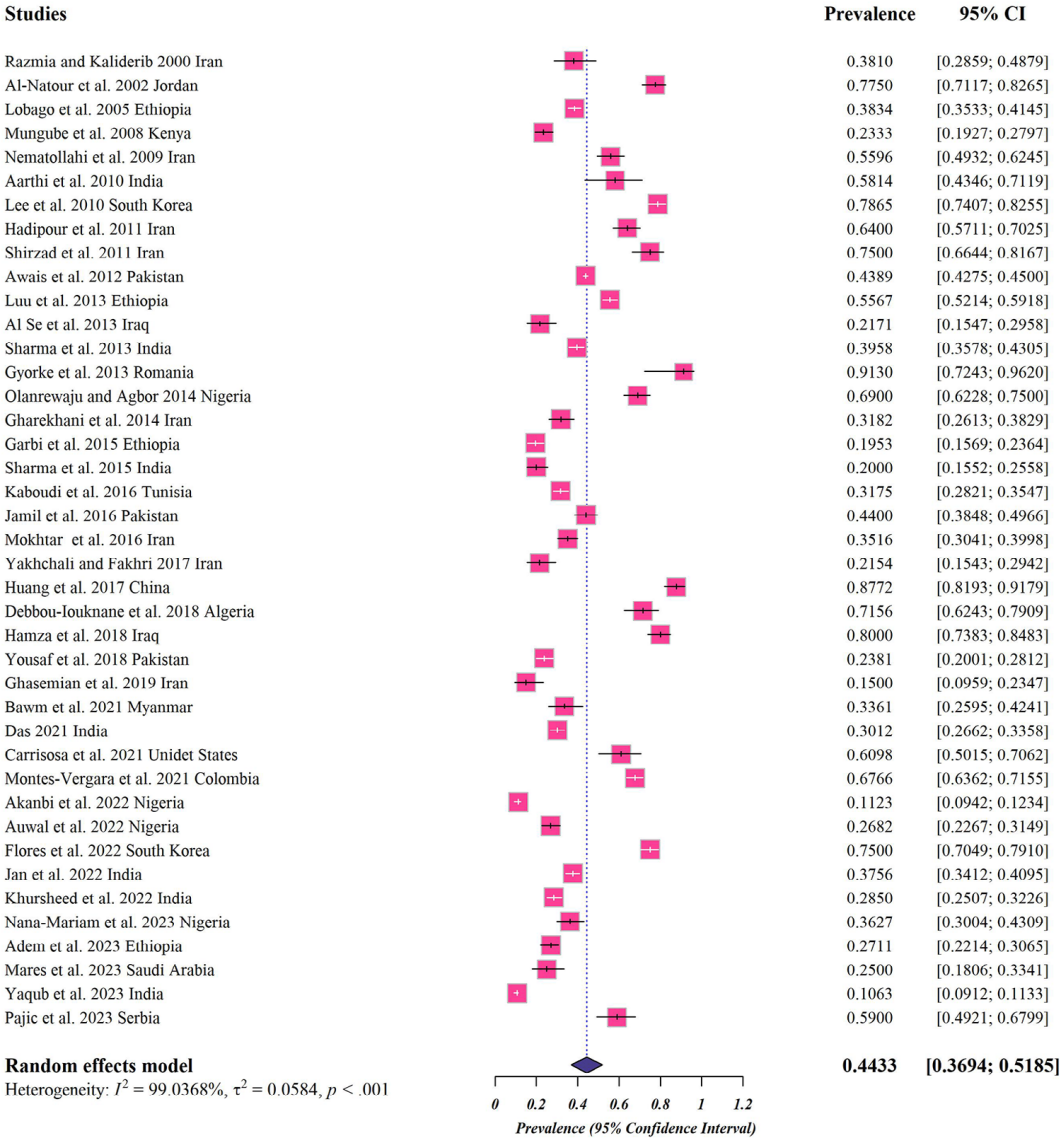
Forest plots for random‐effects meta‐analysis of *Eimeria* infection in domestic chickens based on included studies (The boxes indicate the effect size of the studies (prevalence) and the whiskers indicate its confidence interval for corresponding effect size. There is no specific difference between white and black bars, only studies with a very narrow confidence interval are shown in white. In the case of diamonds, their size indicates the size of the effect, and their length indicates confidence intervals).

A total of 12,870 records were retrieved from databases (651 from Scopus, 946 from PubMed, 372 from ProQuest, 501 from Web of Science and 10,400 from Google Scholar). To determine the eligibility of the literature, a selection process identified 112 full‐text publications. Among these, 8 studies lacked sufficient data, 5 studies presented overlapping data, 9 studies were composed of case reports and case series, and an additional 46 documents did not contain original data, including reviews, letters, theses or workshop materials. Consequently, after a rigorous evaluation based on critical assessment criteria, a total of 41 articles were included in the current meta‐analysis (Figure 1). The estimated pooled global prevalence of *Eimeria* parasites in domestic chickens was 44.3% (95%CI: 36.9%–51.8%) (Figure 2).

The estimations based on WHO regions ranged from 30.8% to 80.5%, which include prevalence rate of 30.8% (95%CI: 21.4%–41.0%) for South‐East Asia Region, 36.3% (95%CI: 24.8%–48.6%) for African Region, 43.2% (95%CI: 31.8%–55%) for Eastern Mediterranean Region, 66% (95%CI: 59.6%–72%) for Region of the Americas, 75.7% (95%CI: 41.2%–98.1%) for European Region and 80.5% (95%CI: 72.6%–87.3%) for Western Pacific Region (Table 2).

Regarding the impact of climate on seasonal variations, our research demonstrates that regions with humid subtropical climate had the highest overall prevalence (75.8%, 95%CI: 46.6%–95.9%) (Table 2).

Moreover, the highest pooled prevalence was observed for an annual precipitation of 300–650 mm (53.6%, 95%CI: 26.5%–79.5%), annual rainfall of 1001–1500 (45.7%, 95%CI: 36.2%–55.4%), average temperature of 10–20°C (53%, 95%CI: 42.6%–63.3%), humidity of <40 (46.7%, 95%CI: 35.1%–58.5%), latitude of >50 (87.7%, 95%CI: 82%–91.9%) and longitude of >110 (76.8%, 95%CI; 72.9%–80.5%) (Table 2).

In terms of season, the highest pooled prevalence rate of *Eimeria* parasites has been found to be related to autumn (58.6%, 95%CI: 40.5%–75.6%) (Table 2).

The analyses based on species or genus were as follows: 38.7% (95%CI: 30.1%–47.7%) for *E. tenella*, 35.5% (95%CI: 24.4%–47.5%) for *E. acervulina*, 21.7% (95%CI: 13.1%–31.8%) for *E. necatrix*, 20.8% (95%CI: 14.2%–28.2%) for *E. maxima*, 18.7% (95%CI: 10.1%–29.1%) for *E. brunetti*, 18.7% (95%CI: 9.9%–29.4%) for *E. mitis*, 14.5% (95%CI: 5.5%–26.4%) for *E. praecox* and 1.4% (95%CI: 0.3%–3%) for *E. mivati* (Figure S1).

In addition, the estimated pooled prevalence based on reports of *Eimeria* spp. in domestic chickens was 31.3% (95%CI: 15.8%–49.2%) (Figure S2).

According to the estimations based on diagnostic techniques, the studies that employed of polymerase chain reaction (PCR) method represented the highest pooled prevalence, with a rate of 68.2% (95%CI: 50.9%–83.1%) (Table 2).

In terms of the type of region (rural/urban), the highest global prevalence of *Eimeria* parasites was observed in samples collected from chickens in urban regions, with a rate of 44.4% (95%CI: 36.5%–52.6%) (Table 2).

### 3.2 Publication bias

**FIGURE 3:**
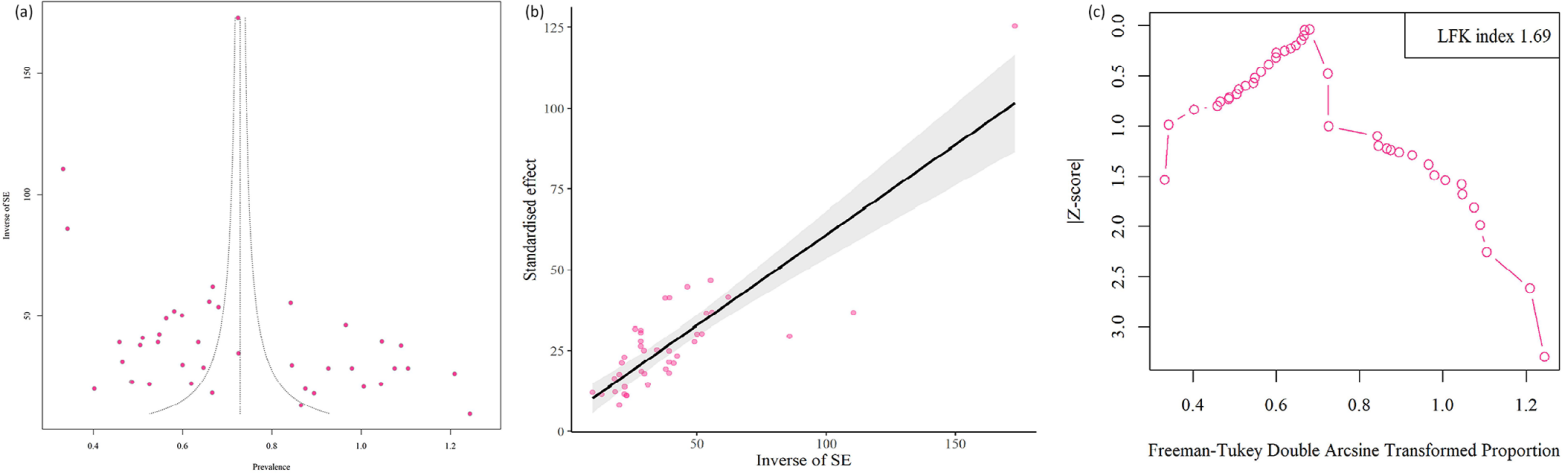
Eggers funnel plot and beggs funnel plot to assess publication bias in *Eimeria* infection in domestic chicken based on included studies (a and b), coloured circles represent each study (coloured circles represent each study. The middle line is the effect size and the other two lines are the corresponding confidence ranges). Doi plot for the global prevalence of *Eimeria* infection in domestic chicken (c), a Luis Furuya‐Kanamori (LFK) index l.69 indicates minor asymmetry.

As revealed by the results of Egger's regression test (*t* = 1.07, *p* = 0.285) and Begg's regression test (*t* = 1.81, *p* = 0.078), there were no significant indications of publication bias. Furthermore, the assessment of the Doi plot unveiled a minor asymmetry (LFK index: 1.69) (Figure 3).

### 3.3 Meta‐regression

**FIGURE 4:**
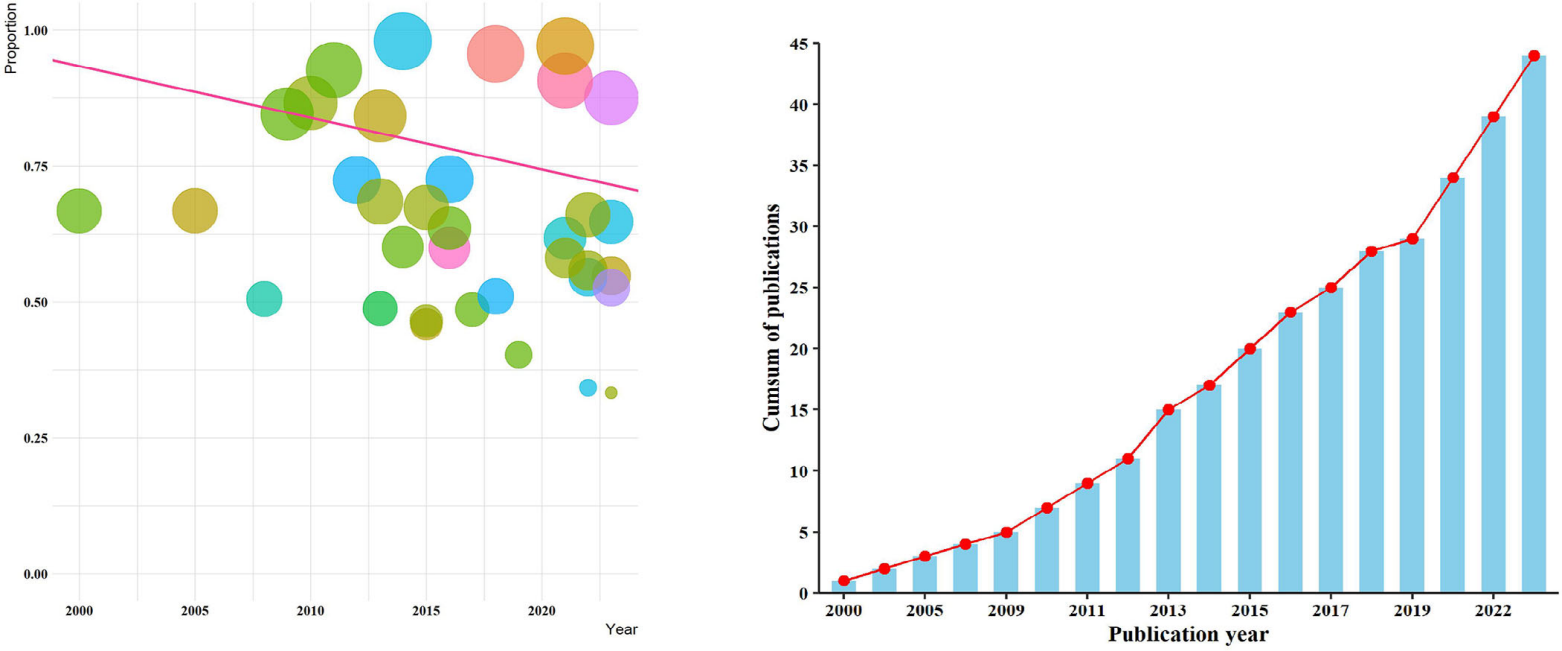
A meta‐regression graph for the global prevalence of *Eimeria* infection in domestic chickens based on year of publication (the pink line is the regression line, which was plotted based on the intercept and the slope of the regression model). The different coloured bubbles represent the countries under study, and their sizes indicate the effect size of each study.

The meta‐regression analysis indicates that there was a statistically significant correlation between prevalence and year of publication (slop: 19.93, *p* < 0.05) (Figure 4).

**FIGURE 5:**
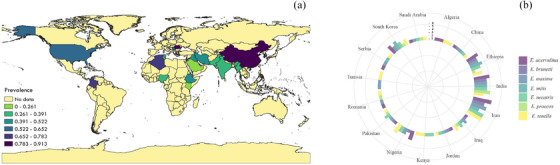
The global prevalence of *Eimeria* infection in domestic chickens in different geographical regions of the world based on included studies (https://qgis.org/en/site/) (a). The circular plot demonstrated the diversity of *Eimeria* species found in the countries under consideration (b).

Based on the included papers, a map was created using the QGIS3 program (available at https://qgis.org/en/site/) to show the prevalence of *Eimeria* infection in domestic chickens in different parts of the world (Figure 5).

## 4 DISCUSSION

Chickens play a crucial role in food production, but the rapid worldwide spread of chicken coccidiosis is a significant threat to the poultry industry (Attree et al., 2021). Epidemiological investigations on the occurrence of *Eimeria* species serve as valuable instruments for the prevention and management of coccidiosis (Morris & Gasser, 2006; Ogedengbe et al., 2011). To the best of our knowledge, this study is the first systematic review and meta‐analysis regarding the prevalence of *Eimeria* infection in domestic chickens.

The results of the current study revealed a global pooled prevalence of 44.3% with *E. tenella* being the most prevalent species (38.7%). Among *Eimeria* parasites affecting chickens, *E. tenella* is regarded as the most critical species due to its high virulence, causing severe haemorrhage and high mortality rates (Fossum et al., 2009). It infects and lives in intestinal epithelial cells, and during its replication cycle, the parasite causes damage to the epithelial cells, which leads to haemorrhagic diarrhoea and negatively impacts growth performance and intestinal health in broiler chickens (Zaman et al., 2012).

The oocysts of *E. tenella* remained viable for 32 days when exposed to 61% humidity and for 49–52 days when subjected to 90% humidity (Fayer, 1980; Gajadhar & Allen, 2004). In the poultry industry, the prevalence of this parasite among chickens resulted in significant economic losses due to expenses associated with prevention and treatment, amounting to over $3.5 billion every year (Blake & Tomley, 2014). Numerous studies have demonstrated that the adverse impacts of infection caused by *E. tenella* in broiler chickens are associated with alterations in the cecal microbiota (Macdonald et al., 2017; Martynova‐Van Kley et al., 2012; Zhou, et al., 2020a).

Nevertheless, even non‐pathogenic species of *Eimeria* have economic significance as their infections can lead to notable declines in feed efficiency, weight gain and overall productivity, even in apparently healthy birds (Morris et al., 2007).

This review highlights that *Eimeria* infections in domestic chickens are most prevalent in regions characterized by a humid subtropical climate, consistent with previous reports indicating that these parasites are endemic in many tropical and subtropical areas worldwide. The presence of a humid and temperate climate provides favourable conditions for the development and persistence of protozoan cysts and/or oocysts in water or the environment (Abebe & Gugsa, 2018; Ahmed et al., 2018).

The optimal conditions for the sporulation of *Eimeria* oocysts are considered to be around 25°C, although these oocysts can endure and remain viable in temperatures as low as 4°C (Anderson et al., 1976; Fayer, 1980).

*Eimeria* oocysts thrive in moist conditions found within poultry houses, which can be due to factors like water spillages or heavy rainfall, leading to humidity levels exceeding 60%. This is particularly relevant in open‐house poultry rearing, a method widely used in tropical and subtropical regions and backyard production setups. In these outdoor settings, when ideal conditions prevail (temperature around 25–30°C, approximately 75% humidity with proper aeration), sporulated oocysts can endure for as long as 602 days (Attree et al., 2021).

Our investigation with regard to different seasons uncovered that the highest occurrence of *Eimeria* infection was in autumn, which can be linked to the preference of oocysts to proceed with the sporulation and its survival during and shortly after rainy seasons in tropical areas. This trend has been observed with a heightened incidence of *Eimeria* infection in regions like Egypt during the winter months (coinciding with the December–February rainy season), Ethiopia following the October rains and the Kashmir valley in India from September to November (Attree et al., 2021).

However, it is worth noting that higher temperatures can have an inhibitory effect, limiting the replication of the parasite. A clear example of this occurrence can be seen in Pakistan, where the highest prevalence of coccidiosis was identified at the end of the monsoon season, a period characterized by a decrease in ambient temperatures to around 25°C (Awais et al., 2012).

The highest pooled prevalence was associated with studies employing PCR technique. The diagnostic approach for coccidiosis typically involves enumerating oocysts in pooled faecal samples or litter, although this method has limitations, such as the restriction on the number of samples processed and the challenging microscopic differentiation of *Eimeria* species. Therefore, the necessity for improved diagnostic efficiency and quality in this regard is an utmost (Velkers et al., 2010). To mitigate the shortcomings of traditional methods, various PCR‐based detection techniques have been developed (Morris et al., 2007; Velkers et al., 2010). One approach involves utilizing quantitative real‐time PCR to identify and quantify various *Eimeria* species in individual samples allowing the assessment of stored samples over extended periods. Additionally, to differentiate *Eimeria* species, non‐quantitative PCR assays are developed (Morris et al., 2007; Velkers et al., 2010).

In line with expectations, our analyses based on different WHO regions showed that the Western Pacific Region represents the highest prevalence of *Eimeria* infections. This region, with 37 countries, is characterized by a significant incidence of neglected tropical diseases. Despite substantial efforts at mass drug administration, parasitic infections persist as a public health concern in the Western Pacific region, largely due to the challenging environmental conditions prevalent in the area (Eslahi et al., 2022).

*Eimeria* infections in chickens are a widespread issue that occurs worldwide. Nevertheless, the prevalence of these infections can greatly differ, and several factors contribute to these variations. These factors may include the population density at which the chickens are raised, the overall health and immune status of the animals, the quality of their nutrition and management practices, the specific breeding techniques employed, the age of the chickens, prevailing climate conditions and the size of the flock (Sun et al., 2023).

The findings from this systematic review and meta‐analysis must be interpreted with caution, considering certain limitations. First, our analyses might have been influenced by publication bias, stemming from either the absence or the limited number of studies available from certain geographical regions. Second, there were various individual case reports related to *Eimeria* species that were not encompassed in the current study. Another limitation is that our study exclusively focused on English‐language publications. Last but not least is that some of the studies included in our analyses exhibited small‐study effects, which can be attributed to factors such as a limited sample size and the absence of a highly sensitive diagnostic technique. Despite these limitations, it is important to acknowledge that this study offers the most comprehensive insights into the prevalence of *Eimeria* infection in domestic chickens from a global standpoint.

## 5 CONCLUSION

Coccidiosis in chickens is a common and potentially serious disease caused by protozoan parasites of the genus *Eimeria*. It can lead to various health issues, including diarrhoea, weight loss, decreased egg production and even death in severe cases. Preventing and managing coccidiosis in poultry is crucial for maintaining the overall health and productivity of the flock. This can be achieved through measures, such as proper hygiene, vaccination and the use of coccidiostats (medications that inhibit the growth of *Eimeria*). Regular monitoring and early intervention are essential to minimize the impact of coccidiosis on chicken populations.

## AUTHOR CONTRIBUTIONS

Aida Vafae Eslahi, Alireza Hayati, Milad Badri and Daniel Diaz: designed the study. Alireza Hayati, Behzad Bijani, Rasoul Samimi, and Aida Vafae Eslahi: Searched for primary publications; screened and appraised primary studies. Alireza Hayati: Extracted the data. Aida Vafae Eslahi, Alireza Hayati, and Milad Badri: Wrote the study manuscript. Meysam Olfatifar, https://www.researchgate.net/profile/Oskar‐Nowak‐2Oskar Nowak, Daniel Diaz, Aida Vafae Eslahi and Milad Badri: Contributed to data analysis and interpretation. Milad Badri, ON, Aida Vafae Eslahi and Daniel Diaz: Edited the manuscript. All authors read the manuscript and participated in the preparation of the final version of the manuscript.

## CONFLICT OF INTEREST STATEMENT

The authors declared no potential conflicts of interest concerning the research or authorship.

## FUNDING INFORMATION

Medical Microbiology Research Center, Qazvin University of Medical Sciences, Qazvin, Iran, Contract No: IR.QUMS.REC.1402.042

## ETHICS STATEMENT

Ethical approval was required and provided for this study, as stated by our institutional review board.

### PEER REVIEW

The peer review history for this paper is available at https://publons.com/publon/10.1002/vms3.1469.

## Supporting information

Supporting Information

Supporting Information

Supporting Information

## Data Availability

All data are included in the manuscript or as supplementary files.
