# Supplementary material for: The global prevalence and associated risk factors of Eimeria infection in domestic chickens: A systematic review and meta‐analysis
Source: Vet Med Sci. 2024 May 30;10(4):e1469. doi: 10.1002/vms3.1469 (PMC11138244; doi:10.1002/vms3.1469)
Supplement: Supplementary file 2 — Supporting Information [file VMS3-10-e1469-s002.pdf]

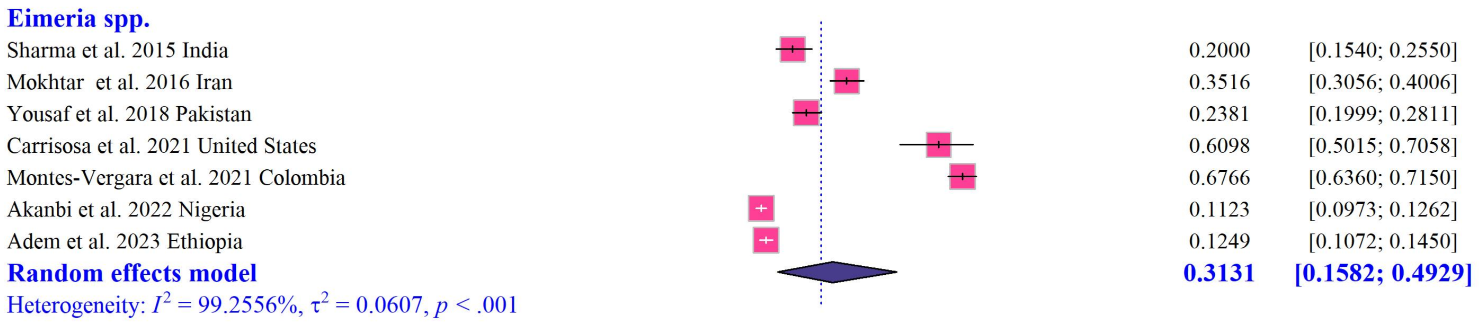

Supplementary Figure 1. Forest plots for random-effects meta-analysis of the global prevalence of *Eimeria* spp. in domestic chickens based on included studies (The boxes indicate the effect size of the studies (prevalence) and the whiskers indicate its confidence interval for corresponding effect size. There is no specific difference between white and black bars, only studies with a very narrow confidence interval are shown in white. In the case of diamonds, their size indicates the size of the effect, and their length indicate confidence intervals).
