# Supplementary material for: The global prevalence and associated risk factors of Eimeria infection in domestic chickens: A systematic review and meta‐analysis
Source: Vet Med Sci. 2024 May 30;10(4):e1469. doi: 10.1002/vms3.1469 (PMC11138244; doi:10.1002/vms3.1469)
Supplement: Supplementary file 3 — Supporting Information [file VMS3-10-e1469-s003.docx]

**Supplementary Table 1.** Quality assessment using the Newcastle–Ottawa scale modified for cross-sectional studies

| No. | First author | Year | Selection^1^  (maximum 5 stars) | Comparability^2^  (maximum 2 stars) | Outcome^3^  (maximum 3 stars) | Total Score |
| --- | --- | --- | --- | --- | --- | --- |
| 1 | Razmia and Kalideri | 2000 | *** | * | ** | 6 |
| 2 | Al-Natour et al. | 2002 | *** | * | ** | 6 |
| 3 | Lobago et al. | 2005 | *** | * | *** | 7 |
| 4 | Mungube et al. | 2008 | *** | * | ** | 6 |
| 5 | Nematollahi et al. | 2009 | *** | ** | *** | 8 |
| 6 | Aarthi et al. | 2010 | *** | * | *** | 7 |
| 7 | Lee et al. | 2010 | *** | * | ** | 6 |
| 8 | Hadipour et al. | 2011 | ** | * | ** | 5 |
| 9 | Shirzad et al. | 2011 | *** | ** | *** | 8 |
| 10 | Awais et al. | 2012 | ** | * | ** | 5 |
| 11 | Gyorke et al. | 2013 | *** | ** | *** | 8 |
| 12 | Al Se et al. | 2013 | *** | * | *** | 7 |
| 13 | Luu et al. | 2013 | *** | ** | *** | 8 |
| 14 | Sharma et al. | 2013 | ** | * | ** | 5 |
| 15 | Gharekhani et al. | 2014 | *** | * | *** | 7 |
| 16 | Olanrewaju and Agbor | 2014 | *** | ** | *** | 8 |
| 17 | Sharma et al. | 2015 | ** | * | ** | 5 |
| 18 | Garbi et al. | 2015 | *** | * | *** | 6 |
| 19 | Mokhtar and Yagoob | 2016 | ** | * | ** | 5 |
| 20 | Kaboudi et al. | 2016 | *** | ** | *** | 8 |
| 21 | Jamil et al. | 2016 | *** | * | ** | 6 |
| 22 | Yakhchali and Fakhri | 2017 | *** | ** | *** | 6 |
| 23 | Huang et al. | 2017 | *** | * | *** | 7 |
| 24 | Debbou-Iouknane et al. | 2018 | ** | ** | *** | 7 |
| 25 | Yousaf et al. | 2018 | *** | * | ** | 6 |
| 26 | Hamza et al. | 2018 | **** | ** | *** | 9 |
| 27 | Ghasemian et al. | 2019 | *** | * | *** | 7 |
| 28 | Montes-Vergara et al. | 2021 | **** | * | *** | 8 |
| 29 | Bawm et al. | 2021 | **** | ** | ** | 8 |
| 30 | Das | 2021 | **** | * | ** | 7 |
| 31 | Carrisosa et al. | 2021 | *** | ** | *** | 8 |
| 32 | Akanbi et al. | 2022 | **** | ** | *** | 9 |
| 33 | Auwal et al. | 2022 | **** | * | *** | 8 |
| 34 | Jan et al. | 2022 | *** | ** | ** | 7 |
| 35 | Khursheed et al. | 2022 | **** | * | *** | 8 |
| 36 | Flores et al. | 2022 | *** | * | *** | 7 |
| 37 | Nana-Mariam et al. | 2023 | *** | ** | *** | 8 |
| 38 | Adem et al. | 2023 | ** | ** | ** | 6 |
| 39 | Yaqub et al. | 2023 | *** | * | *** | 7 |
| 40 | Mares et al. | 2023 | *** | ** | *** | 8 |
| 41 | Pajic et al. | 2023 | **** | * | *** | 8 |

^1^ Selection (Maximum 5 stars): Representativeness of the sample, Sample size, Non-respondents, Ascertainment of the exposure (risk factor). ^2^ Comparability (Maximum 2 stars): The subjects in different outcome groups are comparable, based on the study design or analysis. Confounding factors are controlled. ^3^ Outcome (Maximum 3 stars): Assessment of the outcome, Statistical test
